# Bidirectional association between handgrip strength and ADLs disability: a prospective cohort study

**DOI:** 10.3389/fpubh.2023.1200821

**Published:** 2023-08-17

**Authors:** Senjie Dai, Shihui Wang, Siya Jiang, Dongying Wang, Chenglong Dai

**Affiliations:** ^1^The Second Clinical Medical College, Zhejiang Chinese Medical University, Hangzhou, Zhejiang, China; ^2^The First Clinical Medical College, Zhejiang Chinese Medical University, Hangzhou, Zhejiang, China; ^3^School of Medical Imaging, Hangzhou Medical College, Hangzhou, Zhejiang, China

**Keywords:** handgrip strength, disability, activities of daily living, bidirectional association, CHARLS

## Abstract

**Background:**

Decreased handgrip strength (HGS) and activities of daily living (ADL) disability are common in aging populations. No studies have evaluated the bidirectional associations between HGS and ADL disability. This study aimed to explore the bidirectional effects of HGS and ADL disability.

**Methods:**

This study analyzed data from two waves (2011 and 2015) of China Health and Retirement Longitudinal Study (CHARLS). Low HGS is defined by the Asian Working Group for Sarcopenia criteria. Meanwhile, disability was assessed by ADLs scale. The prospective bidirectional association between HGS and ADL disability was examined using binary logistic regression. Subgroup analysis were performed according to age and gender.

**Results:**

A total of 4,902 and 5,243 participants were included in the Stage I and Stage II analyses, respectively. On the one hand, low HGS was significantly associated with subsequent ADL disability. The odds ratio (OR) value of developing BADL disability and IADL disability were 1.60 (95% confidence interval (CI): 1.23–2.08) and 1.40 (95% CI: 1.15–1.70), respectively, in participants with low HGS. On the other hand, baseline ADL disability was associated with an increased risk of developing low HGS. The OR value of developing low HGS were 1.84 (95% CI: 1.34–2.51) and 1.46 (95% CI: 1.19–1.79) for participants with BADL disability and participants with IADL disability, respectively. Lastly, the strength of the bidirectional associations varied among subgroups.

**Conclusions:**

A significant bidirectional associations were identified between HGS and ADL disability. Interventions should be developed to prevent the development or progression of both low HGS and ADL disability.

## Introduction

Activities of daily living (ADLs) represent the fundamental skills required to care for oneself independently (1). ADL disability is a serious health problem, as a lack of independence increases individuals' vulnerability to their surrounding environment and is associated with poor quality of life (1, 2). Previous studies have shown that ADL disability is strongly associated with an increased risk of depression, falls, healthcare utilization, and death, posing a substantial economic challenge for individuals, families, and society (3–6). As a country with a rapidly aging population, China has a heavy burden of ADL disability. According to one study, older adults (≥65 years old) with ADL disability is projected to increase to 96.2 million by 2060 (7). Therefore, preventing or delaying ADL disability is a public health priority.

Muscle weakness is another important event closely related to aging. Handgrip strength (HGS) has already been demonstrated as a reliable indicator of muscle strength (8). According to numerous studies, low HGS is associated with a range of adverse outcomes, including cognitive impairment, depression, fractures, cardiovascular disease, prolonged hospitalization, and even death (9–11). In addition, low HGS can further accelerate disease progression (12). Therefore, preserving muscle strength is equally important for healthy aging.

Previous studies have investigated the association between HGS and ADL disability. For example, a cross-sectional study in India found that older adults with low HGS had a higher risk of experiencing functional difficulties with ADLs than older adults with strong HGS (13). Similarly, a longitudinal study of 672 Mexican-Americans showed that higher muscle strength was associated with a lower risk of 2-year onset of ADL disability (14). However, previous studies only focused on the unidirectional association between HGS with ADLs disability. At the same time, most existing studies have been limited by small sample sizes or cross-sectional designs. Given the common risk factors and pathophysiological processes underlying HGS and ADL disability, we hypothesize a bidirectional association between the two. Therefore, it is necessary to prospectively explore the bidirectional association between HGS and ADL disability to add new insights into the development of interventions.

In this study, we used data from the China Health and Retirement Longitudinal Study (CHARLS) to (1) investigate the longitudinal association between baseline low HGS and subsequent ADL disability, (2) investigate the longitudinal association between baseline ADL disability and subsequent low HGS; and (3) examine the strength of these associations in different age and gender subgroups.

## Methods

### Study population and data selection

CHARLS is a nationally representative longitudinal survey of middle-aged and older adults in China (15). The baseline survey was conducted in 2011, and three follow-up surveys were conducted in 2013, 2015, and 2018. The study was approved by the institutional review board of Peking University, and all participants provided written informed consent. A detailed description of CHARLS has been published elsewhere (15).

**Figure 1:**
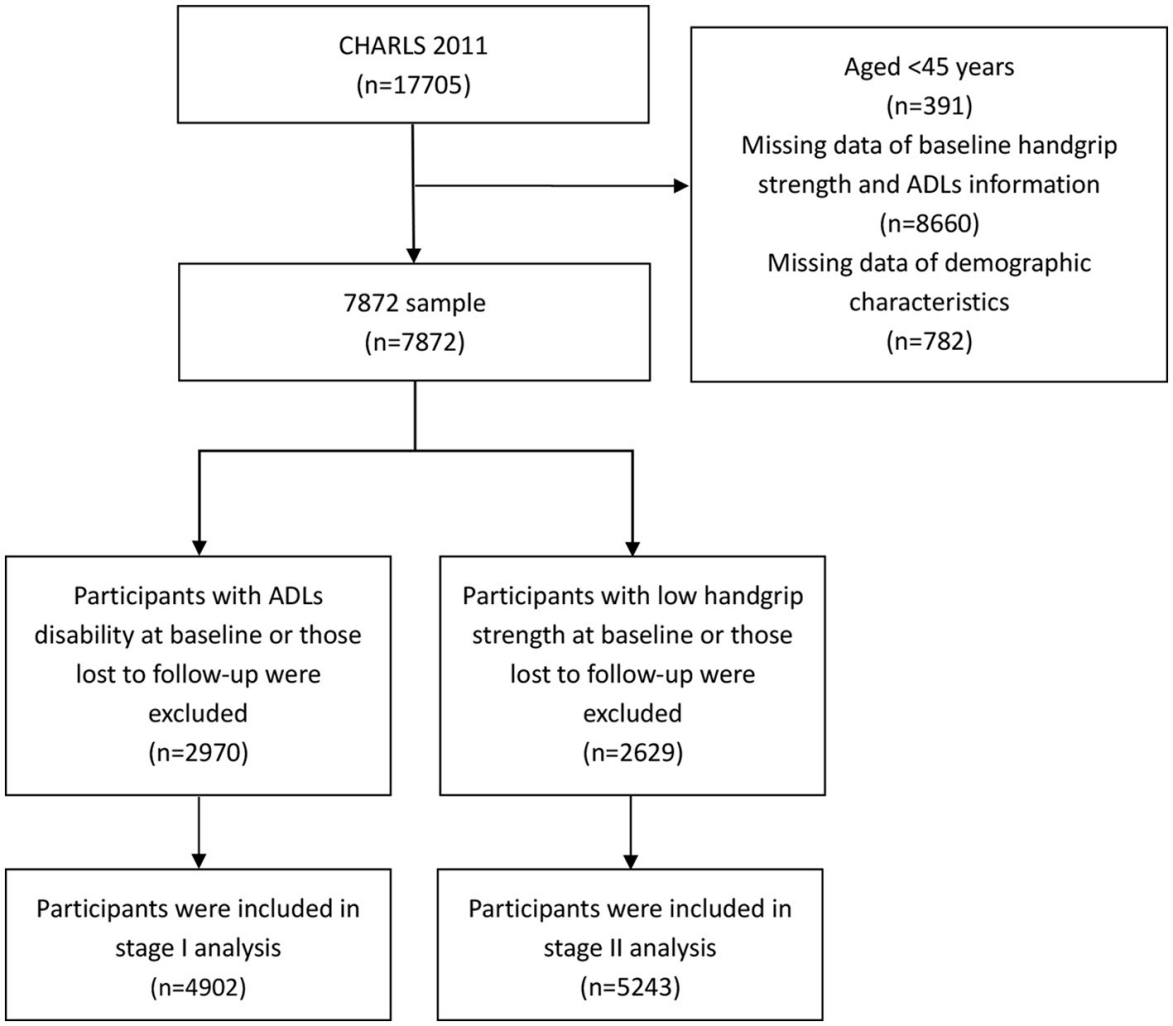
Flowchart on the sample selection.

This current study analyzed CHARLS data obtained from the baseline (2011) and third wave (2015) data collection periods. A total of 9,833 participants were excluded due to (1) age < 45 years, (2) lack of information on baseline HGS and ADLs, (3) lack of information on demographics. In Stage I, after further exclusion of participants with baseline ADL disability and those lost to follow-up, 4,902 participants were included in the analysis. In Stage II, after further exclusion of participants with baseline low HGS and those lost to follow-up, 5,243 participants were included in the analysis. A detailed flowchart is presented in Figure 1.

### Assessment of HGS

HGS was measured using an isometric dynamometer (YuejianTM WL-1000, Nantong, China) (9). Staff demonstrated its proper use prior to testing. Each hand's handgrip strength was tested twice, with the highest value used in the analysis. In accordance with the 2019 guidelines proposed by the Asian Working Group for Sarcopenia (AWGS), 28 kg and 18 kg were defined as cut-off points for low HGS for male and female, respectively (16).

### Assessment of ADL disability

ADL disability was measured using the basic activities of daily living (BADL) and instrumental activities of daily living (IADL) scales (17). BADLs reflected six items measuring dressing, bathing, eating, getting out of bed, toileting, and continence. IADLs consisted of five items, including doing housework, cooking, shopping, taking medication, and handling finances. Each item consisted of four response options (1. no difficulty; 2. difficult but achievable; 3. some difficulties and need help; 4. unable to complete). In accordance with previous studies, outcomes were dichotomized, wherein a lack of complete independence in any BADL/IADL item was defined as having a BADL/IADL disability (4, 18).

### Covariates

Covariates of interest spanned sociodemographic, lifestyle, and health status categories. Sociodemographic characteristics included age, sex, education (primary school and below, middle school, high school and above), address (urban, rural, or other), and marital status (married or other). Lifestyle characteristics: smoking, drinking, sleeping time (< 6 h, 6–8 h, >8 h), napping time (no napping, 0–30 min, 30–60 min, >60 min). In terms of health status, body-mass-index (BMI) was divided into three categories according to Chinese criteria (underweight: ≤ 18.5 kg/m^2^, normal: 18.5–23.9 kg/m^2^, overweight and obese: ≥24 kg/m^2^) (19). Participants' chronic disease status was assessed by self-report and categorized according to the number of chronic diseases (0, 1, 2, ≥3). Falls and fracture history was obtained by asking about falls and fractures over the past 2 years. The Center for Epidemiologic Studies Depression Scale (CES-D-10) was used to screen for the presence of depressive symptoms, with a cutoff score of 10 used to indicate the presence of depressive symptoms (20).

### Statistical analysis

Continuous and categorical variables were presented as means (SD) and counts (percentages), respectively. *T*-tests and Chi-square tests were used to compare baseline characteristics between groups. In Stage I, participants who were able to perform ADLs independently were divided into two groups based on baseline HGS (low vs. normal) and evaluated in 2015 for independence in ADLs. In Stage II, participants with normal handgrip strength were divided into two groups based on presence/absence of ADL disability and evaluated in 2015 for handgrip strength. Binary logistic regression models were constructed to assess the prospective bidirectional association between low HGS and ADL disability. Odds ratios (ORs) and their 95% confidence intervals (CIs) were used to measure the strength of the associations. Multivariate models adjusted for age, sex, education level, address, marital status, smoking, alcohol, BMI, number of chronic diseases, history of falls, history of fracture, night sleep duration, nap duration, and depressive symptoms. In addition, subgroup analyses were performed based on age (≤ 60 years, ≥61 years) and gender. *Post-hoc* power analyses were performed to guide interpretation of our findings (Supplementary Table 1). All analyses were performed in SPSS 25.0. The statistical significance level was set at a 2-sided *P* value < 0.05.

## Results

### Stage I: longitudinal association between baseline low HGS and development of ADL disability

A total of 4,902 participants were included in the no baseline ADL disability cohort. These participants' baseline characteristics are shown in Table 1. The mean age was higher in the low HGS group than in the normal HGS group (*P* < 0.001). Low HGS was also more common in underweight, low education, and other marital status (Separated, divorced, widowed, never married, cohabitation without marriage) groups (*P* < 0.05). Other characteristics associated with low HGS included less sleep, a history of falls, and depression (*P* < 0.05). During the 4-year follow-up period, 104 (14.6%) and 205 (28.8%) participants from the low HGS group developed BADL disability and IADL disability, respectively. As shown in Table 2, participants with low HGS had a significantly increased risk of BADL and IADL disability compared to those with normal HGS (BADLs: OR = 2.38, 95% CI: 1.87–3.03; IADLs: OR = 1.98, 95% CI: 1.65–2.38). Associations remained significant after adjusting for all covariates (BADLs: OR = 1.60, 95% CI: 1.23–2.08; IADLs: OR = 1.40, 95% CI: 1.15–1.70).

**Table 1:** Characteristics of the study population at baseline in Stage I and Stage II.

**Characteristic**	**Stage I (*****N** =* **4,902)**^**a**^			**Stage II (*****N** =* **5,243)**^**b**^					
	**Low HGS**			**BADLs disability**			**IADLs disability**		
	**Yes (*****N** =* **712)**	**No (*****N** =* **4,190)**	***P*** **value**	**Yes (*****N** =* **235)**	**No (*****N** =* **5,008)**	***P*** **value**	**Yes (*****N** =* **721)**	**No (*****N** =* **4,522)**	***P*** **value**
Age	66.12 ± 9.68	59.24 ± 8.87	< 0.001	62.89 ± 9.14	59.08 ± 8.67	< 0.001	61.54 ± 8.85	58.89 ± 8.65	< 0.001
**Sex** ***N*** **(%)**			0.059			0.120			< 0.001
Male	296 (41.6)	1,586 (37.9)		105 (44.7)	1,983 (39.6)		237 (32.9)	1,851 (40.9)	
Female	416 (58.4)	2,604 (62.1)		130 (55.3)	3,025 (60.4)		484 (67.1)	2,671 (59.1)	
**BMI category** ***N*** **(%)**			< 0.001			0.321			0.212
Underweight	90 (12.6)	270 (6.4)		18 (7.7)	321 (6.4)		57 (7.9)	282 (6.2)	
Normal	398 (55.9)	2,052 (49.0)		106 (45.1)	2,502 (50.0)		359 (49.8)	2,249 (49.7)	
Overweight or obesity	224 (31.5)	1,868 (44.6)		111 (47.2)	2,185 (43.6)		305 (42.3)	1,991 (44.0)	
**Address N (%)**			0.057			0.019			0.002
Urban areas	56 (7.9)	447 (10.7)		8 (3.4)	423 (8.4)		40 (5.5)	391 (8.6)	
Rural areas	569 (79.8)	3,207 (76.5)		201 (85.5)	3,997 (79.8)		612 (84.9)	3,586 (79.3)	
Other	87 (12.2)	536 (12.8)		26 (11.1)	588 (11.7)		69 (9.6)	545 (12.1)	
**Education** ***N*** **(%)**			< 0.001			0.681			< 0.001
Primary school or below	615 (86.4)	3,110 (74.2)		180 (76.6)	3,746 (74.8)		619 (85.9)	3,307 (73.1)	
Middle school	65 (9.1)	772 (18.4)		42 (17.9)	914 (18.3)		76 (10.5)	880 (19.5)	
High school or above	32 (4.5)	308 (7.4)		13 (5.5)	348 (6.9)		26 (3.6)	335 (7.4)	
**Marital status** ***N*** **(%)**			< 0.001			0.860			0.249
Married	551 (77.4)	3,634 (86.7)		207 (88.1)	4,392 (87.7)		623 (86.4)	3,976 (87.9)	
Other	161 (22.6)	556 (13.3)		28 (11.9)	616 (12.3)		98 (13.6)	546 (12.1)	
**Smoke** ***N*** **(%)**			0.713			< 0.001			0.004
Still	194 (27.3)	1,082 (25.9)		61 (26.2)	1,353 (27.1)		162 (22.7)	1,252 (27.7)	
Quit	55 (7.7)	333 (8.0)		38 (16.3)	409 (8.2)		77 (10.8)	370 (8.2)	
Never	461 (64.9)	2,769 (66.2)		134 (57.5)	3,235 (64.7)		476 (66.6)	2,893 (64.1)	
**Alcohol** ***N*** **(%)**			0.418			0.854			< 0.001
More than once a month	133 (18.7)	854 (20.4)		51 (21.7)	1,090 (21.8)		126 (17.5)	1,015 (22.4)	
Less than once a month	46 (6.5)	299 (7.1)		14 (6.0)	345 (6.9)		30 (4.2)	329 (7.3)	
Never	533 (74.9)	3,037 (72.5)		170 (72.3)	3,573 (71.3)		565 (78.4)	3,178 (70.3)	
**Night sleep duration (hour)** ***N*** **(%)**			0.047			0.228			0.005
< 6	420 (60.1)	2,280 (55.1)		137 (59.8)	2,673 (54.0)		418 (59.7)	2,392 (53.4)	
6–8	233 (33.3)	1,57 (37.6)		77 (33.6)	1,900 (38.4)		230 (32.9)	1,747 (39.0)	
>8	46 (6.6)	304 (7.3)		15 (6.6)	374 (7.6)		52 (7.4)	337 (7.5)	
**Nap duration (min)** ***N*** **(%)**			0.254			0.681			0.811
No napping	318 (45.2)	1,995 (47.9)		105 (45.7)	2,359 (47.4)		338 (47.9)	2,126 (47.2)	
< 30	124 (17.6)	724 (17.4)		35 (15.2)	842 (16.9)		123 (17.4)	754 (16.8)	
30–60	171 (24.3)	878 (21.1)		57 (24.8)	1,079 (21.7)		154 (21.8)	982 (21.8)	
>60	91 (12.9)	568 (13.6)		33 (14.3)	697 (14.0)		91 (12.9)	639 (14.2)	
**Number of chronic diseases** ***N*** **(%)**			0.748			< 0.001			< 0.001
0	162 (22.8)	1,028 (24.5)		20 (8.5)	1,266 (25.3)		116 (16.1)	1,170 (25.9)	
1	217 (30.5)	1,272 (30.4)		58 (24.7)	1,538 (30.7)		183 (25.4)	1,413 (31.2)	
2	171 (24.0)	958 (22.9)		53 (22.6)	1,116 (22.3)		178 (24.7)	991 (21.9)	
≥3	162 (22.8)	932 (22.2)		104 (44.3)	1,088 (21.7)		244 (33.8)	948 (21.0)	
**History of falls N (%)**			< 0.001			< 0.001			< 0.001
Yes	164 (23.2)	711 (17.1)		75 (32.6)	932 (18.7)		225 (31.8)	782 (17.4)	
No	543 (76.8)	3,459 (82.9)		155 (67.4)	4,048 (81.3)		482 (68.2)	3,721 (82.6)	
**History of fracture** ***N*** **(%)**			0.569			< 0.001			< 0.001
Yes	14 (2.0)	70 (1.7)		13 (5.7)	88 (1.8)		27 (3.8)	74 (1.6)	
No	693 (98.0)	4,099 (98.3)		216 (94.3)	4,892 (98.2)		679 (96.2)	4,429 (98.4)	
**Depressive symptom** ***N*** **(%)**			< 0.001			< 0.001			< 0.001
Yes	381 (53.5)	1,905 (45.5)		165 (70.2)	2,63 (55.2)		478 (66.3)	1,932 (42.7)	
No	331 (46.5)	2,285 (54.5)		70 (29.8)	2,245 (44.8)		243 (33.7)	2,590 (57.3)	

Results are presented as mean ± SD, or n (%). *P* < 0.05 was considered statistically significant. BMI, body mass index; HGS, handgrip strength; BADLs, basic activities of daily living; IADLs, instrumental activities of daily living. ^a^Missing data: 8 for smoke, 62 for night sleep duration, 33 for Nap duration, 25 for History of falls, 26 for History of fracture. ^b^Missing data: 13 for smoke, 67 for night sleep duration, 36 for Nap duration, 33 for History of falls, 34 for History of fracture.

**Table 2:** Logistic regression of handgrip strength for ADLs.

	**BADLs**		**IADLs**	
	**OR (95% CI)**	***P*** **value**	**OR (95% CI)**	***P*** **value**
**Crude model**				
Normal HGS	1		1	
Low HGS	2.38 (1.87–3.03)	< 0.001	1.98 (1.65–2.38)	< 0.001
**Model 1**				
Normal HGS	1		1	
Low HGS	1.60 (1.23–2.08)	< 0.001	1.40 (1.15–1.70)	0.001

OR, odds ratio; 95% CI, 95% confidence interval; HGS, handgrip strength; BADLs, basic activities of daily living; IADLs, instrumental activities of daily living. Model 1 adjusted the following variables: age, sex, BMI, address, education, marital status, smoke, alcohol, night sleep duration, nap duration, number of chronic diseases, history of falls, history of fracture, depression.

### Stage II: longitudinal association between baseline ADL disability and development of low HGS

At this stage, 5,243 individuals were included for analysis. Compared with individuals without ADL disability, those with functional disabilities were older and more likely to live in rural areas (*P* < 0.05). In terms of health status, individuals with ADL disability had a higher number of chronic diseases, a history of falls or fractures, and depressive symptoms (*P* < 0.05). The detailed baseline characteristics of individuals with and without ADL disability are presented in Table 1. During the 4-year follow-up period, 77 (32.8%) participants with BADL disability and 193 (26.8%) participants with IADL disability developed low HGS. Logistic regression suggested that participants with BADL disability or IADL disability had a higher risk of low HGS compared to individuals with normal function (BADLs: OR = 2.52, 95% CI: 1.90–3.35; IADLs: OR = 2.01, 95% CI: 1.68–2.42). After adjusting for all confounders, the association remained statistically significant (BADLs: OR = 1.84, 95% CI: 1.34–2.51; IADLs: OR = 1.46, 95% CI: 1.19–1.79). Detailed results are presented in Table 3.

**Table 3:** Logistic regression of ADLs disability for HGS.

	**Low HGS**	
	**OR (95%CI)**	***P*** **value**
**Crude model**		
BADLs	1	
BADLs	2.52 (1.90–3.35)	< 0.001
**Model 1**		
BADLs	1	
BADLs	1.84 (1.34–2.51)	< 0.001
**Crude model**		
IADLs	1	
IADLs	2.01 (1.68–2.42)	< 0.001
**Model 1**		
IADLs	1	
IADLs	1.46 (1.19–1.79)	< 0.001

OR, odds ratio; 95% CI, 95% confidence interval; HGS, handgrip strength; BADLs, basic activities of daily living; IADLs, instrumental activities of daily living. Model 1 adjusted the following variables: age, sex, BMI, address, education, marital status, smoke, alcohol, night sleep duration, nap duration, number of chronic diseases, history of falls, history of fracture, and depression.

### Subgroup analysis

**Figure 2:**
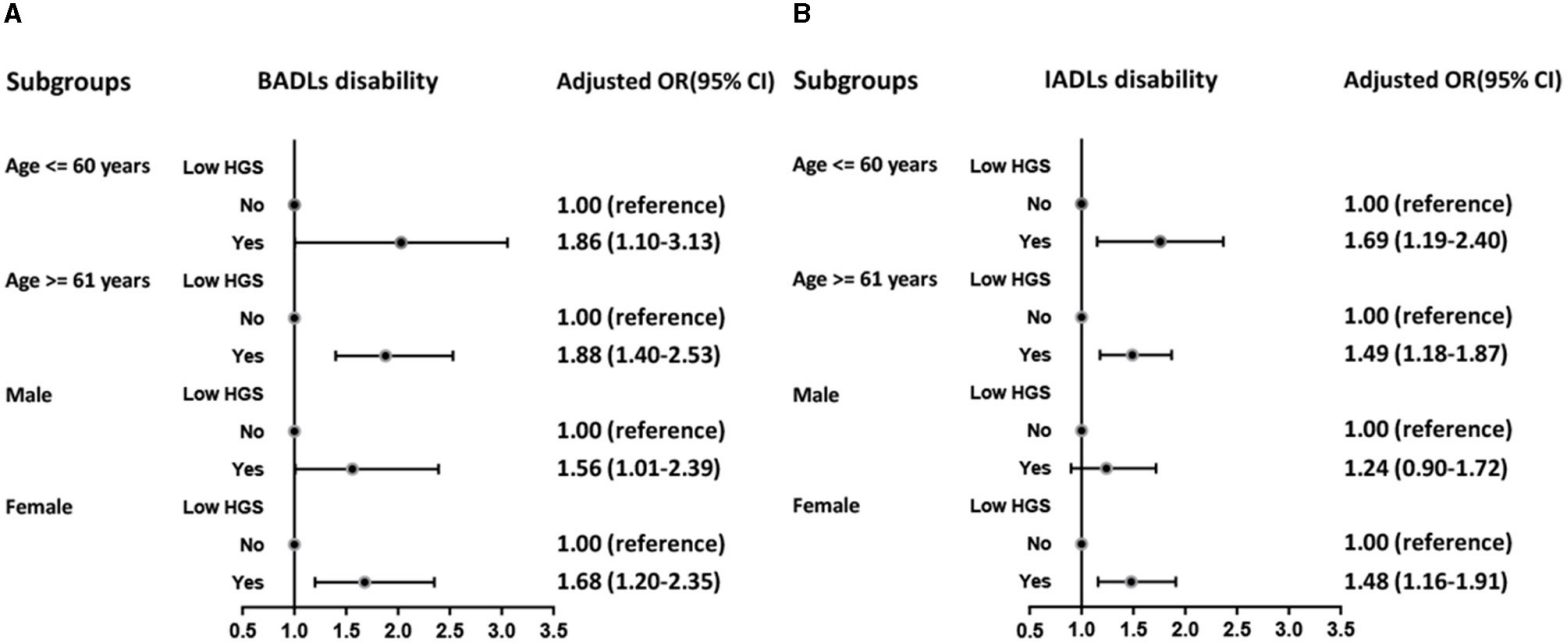
Logistic regression analysis of low HGS for BADLs disability **(A)** and IADLs disability **(B)** among subgroups.

Age and sex subgroup analyses were conducted for both analysis stages. As shown in Figure 2, women with low HGS had a higher risk of ADL disability than men with low HGS (BADLs: OR = 1.68, 95% CI: 1.20–2.35; IADLs: OR = 1.48, 95% CI: 1.16–1.91). Further, participants with low HGS that were aged < 60 years had a higher risk of IADL disability (OR = 1.69, 95% CI: 1.19–2.40). In contrast, the risk of BADL disability did not significantly differ by age subgroups.

**Figure 3:**
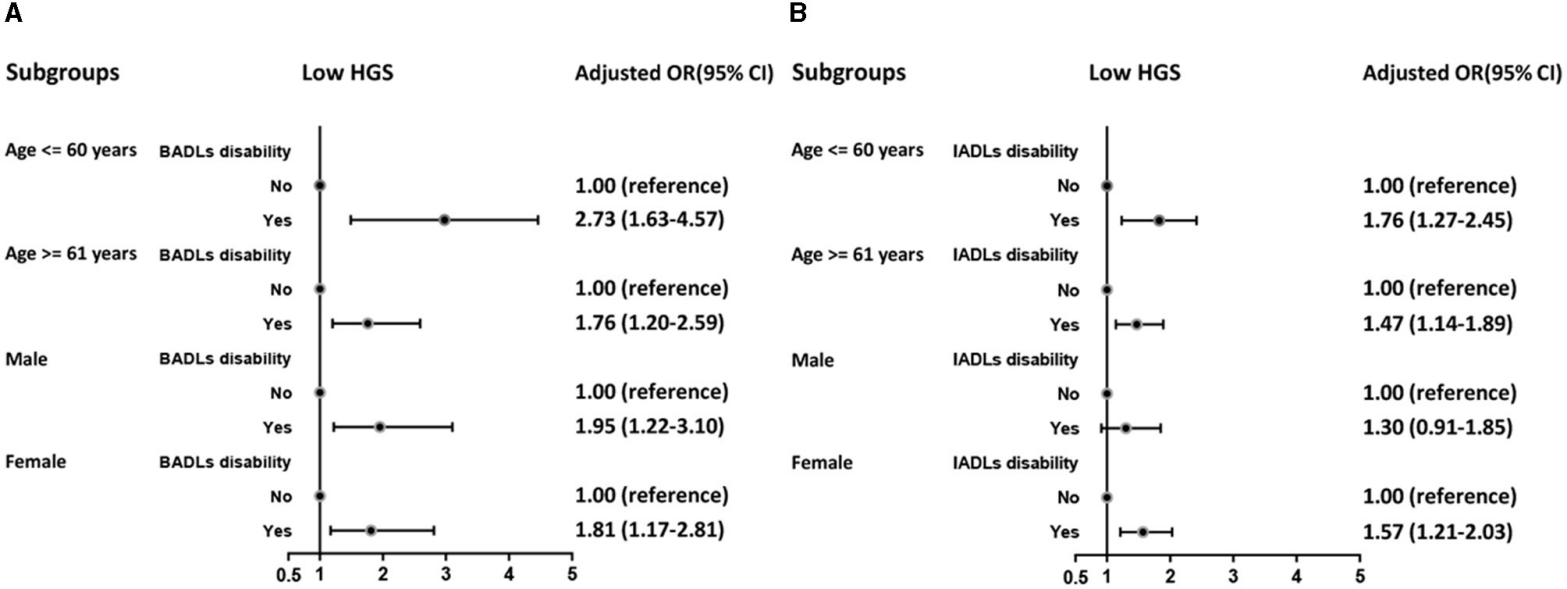
Logistic regression analysis of BADLs disability **(A)** and IADLs disability **(B)** for low HGS among subgroups.

Figure 3 displays the association between ADL disability and the development of low HGS among subgroups defined by age and sex. The results showed that men with BADL disability (OR = 1.95, 95% CI: 1.22–3.10) and women with IADL disability (OR = 1.57, 95% CI: 1.21–2.03) were at higher risk of developing low HGS. In addition, the risk of developing low HGS was higher in the aged ≤ 60-year subgroup, both in those with BADL dysfunction (OR = 2.73, 95% CI: 1.63–4.57) and IADL dysfunction (OR = 1.76, 95% CI: 1.27–2.45).

## Discussion

This study determined that a bidirectional association exists between low HGS and ADL disability. Specifically, low HGS was found to predict the development of ADL disability, and ADL disability increased the risk of developing low HGS. These associations remained significant even after adjusting for confounding factors. Besides, the association between HGS and ADL disability varied by age and sex.

Previous studies have focused on the effect of HGS on ADL disability. One Australian cross-sectional study found that for every 10 kg increase in HGS, the odds of ADL impairment decreased by 39% (21). Similarly, low HGS was associated with an increased risk of all ADL limitations in an 8-year longitudinal study (22). In addition, a prospective study involving 555 participants determined that low HGS predicted accelerated dependence in ADLs (23). In the present study, after adjusting for multiple variables, low HGS was strongly associated with subsequent ADL disability, thereby affirming the reliability of HGS in identifying those at risk of ADL disability. At the same time, the goal of preventing the development of ADL disability is achievable given the non-invasiveness and simplicity of HGS testing and the effectiveness of exercise to improve muscle strength (24). Therefore, early monitoring and intervention of HGS is an important approach to delaying the progression of ADL disability, and it is necessary to include HGS as a routine screening tool for ADL disability. Notably, HGS was more strongly associated with BADLs than IADLs in this study. Generally, IADL disability occurs prior to BADL disability as it reflects more complex functional activity (25). According to previous research, cognition may play a more dominant role in IADLS, which may explain why HGS is more strongly associated with BADL disability than IADL disability (26).

To the best of our knowledge, no study has investigated the effect of ADL disability on HGS. However, previous studies have investigated the effect of physical activity on HGS, wherein a lack of physical activity was found to lead to a decrease in HGS (27, 28). In addition, a study noted a higher incidence of sarcopenia in older adults with functional limitations (29). In this study, ADL disability increased the risk of low HGS. In addition, BADL disability was more closely associated with the development of low HGS than IADL disability. This is understandable as BADLs reflect simpler activities. Thus, BADL disability makes an individual more susceptible to the condition of their surrounding environment. In sum, our findings broaden the evidence in the existing field that there may be a mutually exacerbating process between low HGS and ADLs disability.

The mechanisms that underlie the bidirectional association between HGS and ADL disability are likely complex and diverse. On the one hand, individuals with low HGS usually have higher levels of inflammation (30). Thus, inflammation may be an intermediary bridge between low HGS and ADL disability (30, 31). In addition, pain was found to be a co-existing factor in low HGS, resulting in limited ADL activity (32). On the other hand, individuals with ADL disability experience difficulties with functional activities like eating and shopping, which may result in insufficient nutritional intake and declines in muscle strength. Moreover, people with ADL dysfunction usually experience psychological disorders, mental distress, and cognitive decline, all of which are thought to be closely associated with HGS decline (33, 34). In fact, ADL disability is also a stress state, and long-term stress changes can lead to muscle strength weakening (33). Finally, common risk factors and unhealthy lifestyles may also be important causes of this association. For example, individuals with low HGS and ADL disability have significantly decreased interest and frequency in participating in physical activity, accelerating ADL disability progression and muscle strength decline (35, 36).

In terms of subgroups, we found a stronger association between low HGS and subsequent ADL disability in women, which is supported by previous findings (13, 37). It has been shown that women have less muscle strength and mass than men and are more sensitive to changes in muscle composition (37). Women also have lower levels of androgens, which play an important role in skeletal muscle repair (38). Gender differences in physical activity may also be an explanation for this phenomenon. Specifically, men may be more frequently physically active, while women are less actively engaged in exercise, leading to an accelerated decline in HGS (39, 40). Previous studies have found a higher incidence of disability among Chinese women, which may be partly explained by the gender differences in the above associations (41). Other than for the effect of low HGS on BADLs (which was similar across age groups), the strength of the associations was higher in the younger age group. We speculate that this may be due to an increased proportion of other contributing factors, such as chronic diseases in the older age group, resulting in a lower proportion of HGS interacting with ADL disability (42). Taken together, the above findings highlight the need to consider age and gender when developing intervention strategies.

### Strengths and limitations

This is the first prospective study to investigate the bidirectional association between low HGS and ADL disability. The study's strengths include a large nationally representative sample and longitudinal study design. However, some limitations present in the study should also be considered. First, some of the data were obtained through self-report, introducing the potential for recall bias. Second, the study design of CHARLS prevented us from obtaining multiple assessments of ADL disability status, which may be interfered with intermittent disability. Third, due to the limited number of individuals with ADL disability, the influence of specific ADLs on HGS is unknown and warrants exploration through future high-quality studies. Finally, although the overall sample was relatively large, a large number of participants were excluded due to a loss to follow-up and missing data, which may have resulted in selection bias.

## Conclusion

In conclusion, this study suggested a bidirectional association between HGS and ADL disability. Low HGS can be used as a reliable marker of future ADL disability, which in turn exacerbates the decline in HGS. It is necessary to strengthen the screening and intervention of low HGS and enhance the functional recovery of individuals with ADL disability to promote healthy aging. Moreover, age and gender should be considered when assessing the association between HGS and ADL disability to develop more accurate and effective interventions.

## Data availability statement

The original contributions presented in the study are included in the article/Supplementary material, further inquiries can be directed to the corresponding author.

## Ethics statement

The studies involving humans were approved by Biomedical Ethics Review Committee of Peking University. The studies were conducted in accordance with the local legislation and institutional requirements. The participants provided their written informed consent to participate in this study. No potentially identifiable images or data are presented in this study.

## Author contributions

SD: conceptualization and writing. SW: methodology. SJ: validation. DW: software. CD: investigation. All authors read and approved the final manuscript.
