# Supplementary material for: Bidirectional association between handgrip strength and ADLs disability: a prospective cohort study
Source: Front Public Health. 2023 Aug 17;11:1200821. doi: 10.3389/fpubh.2023.1200821 (PMC10470652; doi:10.3389/fpubh.2023.1200821)
Supplement: Supplementary file 1 [file Table_1.docx]

**Supplementary Table 1-1** **Post-hoc power analysis for BADLs and IADLs**

| Parameters | BADLs | IADLs |
| --- | --- | --- |
| Sample size | 4902 | 4902 |
| $\alpha$ | 0.05 | 0.05 |
| P0 | 6.7% | 16.9% |
| P1 | 14.6% | 28.8% |
| R^2^ | 0.133 | 0.133 |
| Percent | 14.5% | 14.5% |
| Power | 99.99% | 100% |

BADLs, basic activities of daily living. IADLs, instrumental activities of daily living.

P0 represents the probability of BADLs or IADLs disability with normal grip strength, respectively; P1 represents the probability of BADLs or IADLs disability with low grip strength, respectively; R^2^ represents the determinants of grip strength grade and other corrective confounding factors; Percent indicates the percentage of people with low grip strength.

**Supplementary Table 1-2** **Post-hoc power analysis for Low HGS**

| Parameters | Low HGS ^a^ | Low HGS ^b^ |
| --- | --- | --- |
| Sample size | 5243 | 5243 |
| $\alpha$ | 0.05 | 0.05 |
| P0 | 16.2% | 15.4% |
| P1 | 32.8% | 26.8% |
| R^2^ | 0.124 | 0.073 |
| Percent | 4.5% | 13.8% |
| Power | 99.97% | 100% |

HGS, handgrip strength.

a: independent variable is BADLs; b: independent variable is IADLs.

P0 represents the probability of low grip strength with normal BADLs or IADLs function, respectively; P1 represents the probability of low grip strength with BADLs disability or IADLs disability, respectively; R^2^ represents the determinants of BADLs or IADLs grade and other corrective confounding factors; Percent indicates the percentage of people with BADLs disability or IADLs disability.
